# Association of Leukotriene A4 Hydrolase with Tuberculosis Susceptibility Using Genomic Data in Portugal

**DOI:** 10.3390/microorganisms7120650

**Published:** 2019-12-04

**Authors:** Teresa Rito, Joana Ferreira, Bruno Cavadas, Pedro Soares, Olena Oliveira, Martin B. Richards, Raquel Duarte, Luísa Pereira, Margarida Correia-Neves

**Affiliations:** 1 Life and Health Sciences Research Institute (ICVS), School of Medicine, University of Minho, 4710-057 Braga, Portugal; id7080@alunos.uminho.pt (O.O.); mcorreianeves@med.uminho.pt (M.C.-N.); 2 ICVS/3B’s, PT Government Associate Laboratory, Braga/Guimarães, 4805-017 Guimarães, Portugal; 3 Instituto de Investigação e Inovação em Saúde, Universidade do Porto (i3S), 4200-135 Porto, Portugal; joanaf@ipatimup.pt (J.F.); bcavadas@ipatimup.pt (B.C.); luisap@ipatimup.pt (L.P.); 4 Instituto de Patologia e Imunologia Molecular da Universidade do Porto (IPATIMUP), 4200-135 Porto, Portugal; 5 Centre of Molecular and Environmental Biology (CBMA), Department of Biology, University of Minho, 4710-057 Braga, Portugal; pedrosoares@bio.uminho.pt; 6 Institute of Science and Innovation for Bio-Sustainability (IB-S), University of Minho, 4710-057 Braga, Portugal; 7 EPI Unit, Institute of Public Health University of Porto (ISPUP), 4050-091 Porto, Portugal; rdmelo@med.up.pt; 8 Department of Biological and Geographical Sciences, School of Applied Sciences, University of Huddersfield, Huddersfield HD1 3DH, UK; M.B.Richards@hud.ac.uk; 9 Clinical Epidemiology, Predictive Medicine and Public Health Department, Faculty of Medicine, University of Porto, 4200-319 Porto, Portugal

**Keywords:** Tuberculosis (TB) susceptibility, eicosanoids, gene polymorphisms, genomics, infectious diseases

## Abstract

Leukotriene A4 hydrolase (LTA4H) is a key enzyme in the eicosanoid pathway. *lta4h* locus polymorphisms have previously been linked to tuberculosis (TB) susceptibility and disease outcome in a Vietnamese dataset, but further studies suggested that those results were poorly reproducible. We, therefore, compared the full set of variants (113 SNPs) within the gene in a Portuguese dataset of 112 TB patients and 120 controls, using both the frequency of SNPs and haplotypes, in order to assess their association with TB susceptibility. Although we obtained no significant differences between the TB patients and the control group, linkage analysis showed that an extensively typed polymorphism, *rs17525495*, was associated with 21 other SNPs, all displaying evidence of association to lower LTA4H expression. While the derived alleles of these SNPs showed a moderately higher frequency in the TB group, differences were not significant. In contrast to Asian populations, where these SNPs are much more frequent, the low frequencies of candidate SNPs in Europeans render them less pertinent in a public health context. Consequently, the typing of specific polymorphisms as a strategy to establish preventive measures and differential TB drug treatments is important but needs to take into consideration that haplotypic background and structure can be substantially different in distinct geographic regions.

## 1. Introduction

Eicosanoids are host lipids that have been shown to act as tuberculosis (TB) susceptibility modulators. They regulate inflammation [1] by mediating both pro- or anti-inflammatory responses, ultimately influencing the nature and magnitude of T-cell and cytokine responses. The dual inflammatory action is, in turn, dependent on the activity of leukotriene A4 hydrolase (LTA4H), an enzyme that converts leukotriene A4 (LTA4) into leukotriene B4 (LTB4). LTB4 recruits several immune cells, including neutrophils-key players in inflammation. However, it also mediates the degradation of the neutrophilic chemoattractant PGP (proline-glycine-proline), decreasing the recruitment of neutrophils, and potentially thereby generating anti-inflammatory responses [2].

*lta4h* locus polymorphisms have been controversially associated with susceptibility to *Mycobacterium tuberculosis* (Mtb). Two SNPs (*rs1978331*/*rs2660898*), detected in a Vietnamese cohort [3], were equally linked in this population to both weak and exacerbated inflammatory responses to Mtb in individuals with homozygous genotypes for the different alleles of the SNPs. This might favour heterozygous individuals, where the inflammatory response might be better adjusted. However, no association of these polymorphisms was shown in large Russian, Mozambican, and Chinese cohorts [4,5,6] and heterozygous advantage, as proposed for these SNPs, is an extremely rare phenomenon. Another SNP in the *lta4h* promoter (*rs17525495*) was identified later as altering transcriptional activity [7], increasing TB susceptibility, and it has subsequently been extensively typed.

Very little is known about the overall variation in the gene. To begin to rectify this, we present the first *lta4h* genomic analysis comparing a general population and TB patients. We analysed all variants and linkage patterns throughout the locus instead of a limited number of SNPs, improving our understanding of the role of LTA4H in TB susceptibility in a European context.

## 2. Materials and Methods

Cases were adult TB patients (Mtb culture-positive), confirmed as HIV-negative through serological analysis, collected from outpatient TB centres in northern Portugal in 2014–2015. We diagnosed TB through isolation of Mtb or positive nucleic acid amplification in respiratory or pleural specimens. Controls were volunteers recruited from primary healthcare units in the same region, without previous TB, which we evaluated by IFN-γ release assay (IGRA) to exclude latent TB. We obtained controls and cases from the same cohort under similar TB exposure levels (often non-relative household members of cases). We excluded from the study patients and controls that tested positive for HIV infection, since it drastically increases the probability of contracting TB and other infections.

We considered only individuals autochthonous to Porto in the study, eliminating background statistical noise regarding migrants that could have carried TB from high-incidence TB countries (i.e., with different exposure levels). This also eliminates background statistical noise by stipulating a better-defined common gene pool for both patients and controls. We collected patients’ epidemiological and clinical information, including sex, place of residence (parish), incidence or otherwise of alcohol or drug misuse, smoking habit, co-morbidities (hypertension, asthma, bronchitis, chronic obstructive pulmonary disease (COPD), heart condition, hepatitis, silicosis), HIV status and the aforementioned description of previous TB treatment (used as an exclusion factor). Public health professionals obtained similar information for controls upon recruitment for the study.

This study was carried out in accordance with the recommendations of the Portuguese Northern Region Health Administration Ethics Committee (68/2014), which approved the protocol for this study. All subjects gave written informed consent in accordance with the Declaration of Helsinki.

We purified genomic DNA from 232 samples (112 TB patients, 120 controls) from whole blood using PureLink^®^ (Invitrogen, Carlsbad, CA, USA) and sequenced them using Illumina MiSeq V3 technology with 300 bp paired-end reads for an average coverage of 40×. We sequenced the complete *lta4h* gene, including the promoter and introns (Chromosome 12:96,000,828–96,043,520, build GRCh38), at LGC Genomics Laboratory, Germany. Raw data are publicly available at the European Nucleotide Archive (ENA) with Accession Number PRJEB35651. We carried out the sequence assembly using Burrow–Wheeler aligner bwa (v.0.7.15-r1140) and called SNPs using Genome Analysis Toolkit (GATK) HaplotypeCaller (v3.7-0-gcfedb67).

We compared SNP frequencies and tested haplotype associations using PLINK (v1.90b4.4) (Available online: https://www.cog-genomics.org/plink/1.9/). We tested SNP associations in two ways: One standard case/control association analysis without covariates (by obtaining an asymptotic p-value for a chi-square test between allelic frequencies), and a second, considering covariates in cases and controls. We adjusted an asymptotic *p*-value for a *t*-statistic for the covariates (sex, age, addictions, and co-morbidities) in order to account for differential group structure between cases and controls. The haplotype association test is based on the comparison between the frequency of combined SNPs, in a similar fashion to standard case/control association analysis, following the use of the expectation–maximization (EM) algorithm to detect probable haplotypes.

We performed haplotypic reconstruction and haplotypic composition comparison between groups using PHASE (v.2.1.1) (Available online: http://stephenslab.uchicago.edu/software.html) with its standard parameters. PHASE implements a Bayesian statistical method for reconstructing haplotypes from population genotype data. We also used this software to perform permutation tests for significant differences in haplotype frequencies between case and control groups. We employed Haploview [8] to estimate linkage measures and build linkage maps (using *r*^2^) between SNPs. For comparison with the Portuguese data, we obtained Vietnamese genomic data from the 1000 Genomes Project database [9,10], since Vietnam has been highlighted as relevant in previous studies [3].

We checked SNPs associated with differential LTA4H expression using the GTEx database (Available online: https://www.gtexportal.org/).

## 3. Results

**Figure 1:**
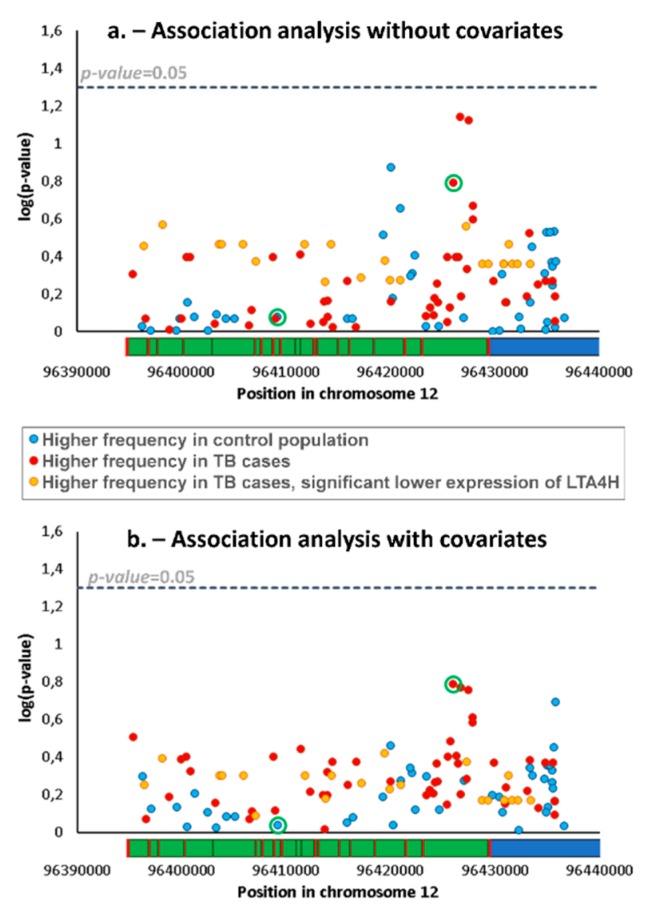
Association analysis between the 113 SNPs in the *lta4h* locus detected in tuberculosis (TB) and control group in a Portuguese population using a standard case/control association analysis (**a**) and an analysis adjusted for covariates (**b**). Green circles indicate two SNPs, *rs1978331* and *rs2660898* from left to right, previously associated with TB susceptibility. The horizontal bar in each graph shows the gene topology of *lta4h* locus, indicating the 5’ untranslated region (blue), exons (red) and introns (green). None of the SNPs analysed was located in exons.

Genomic analysis of *lta4h* in 232 samples (i.e., a total of 464 chromosomes) revealed 331 SNPs, 113 of which had a minimum allele frequency higher than 2% and which therefore qualified for further analyses (Table S1). We compared frequencies of SNPs and haplotypes (i.e., a combination of two/three SNPs) between patients and controls using standard association analyses for the detection of different genetic backgrounds, potentially related to differential TB susceptibility. We obtained no significant difference for any SNP (Figure 1a) or haplotype.

We also performed the association analyses adjusted for covariates. The list of covariates in the TB patients and control group is shown in Table 1. Some of the individual covariates are significantly different between groups and, in general, the TB group includes a significantly higher number of males (*p* < 0.00001), younger individuals (*p* < 0.00001), and proportion of addictions (*p* = 0.00026), but was not significantly different for co-morbidities in general (*p* = 0.05480), most of them reflecting general trends for TB infection in the wider Portuguese population [11]. The refinement of the analysis with covariates did not reveal any significant difference in SNP proportions (Figure 1b).

We also performed phasing of the data (establishing the most probable combination of SNPs across chromosomes) and compared overall haplotypic structure between the two groups using a permutation test in PHASE, confirming no significant difference between the groups (*p* = 0.17). Unlike the previous tests, this does not compare frequencies of specific SNPs or haplotypes but rather considers the differences in the full dataset between both groups under the null hypothesis that the case and control haplotypes are a random sample from a single set of haplotype frequencies.

**Figure 2:**
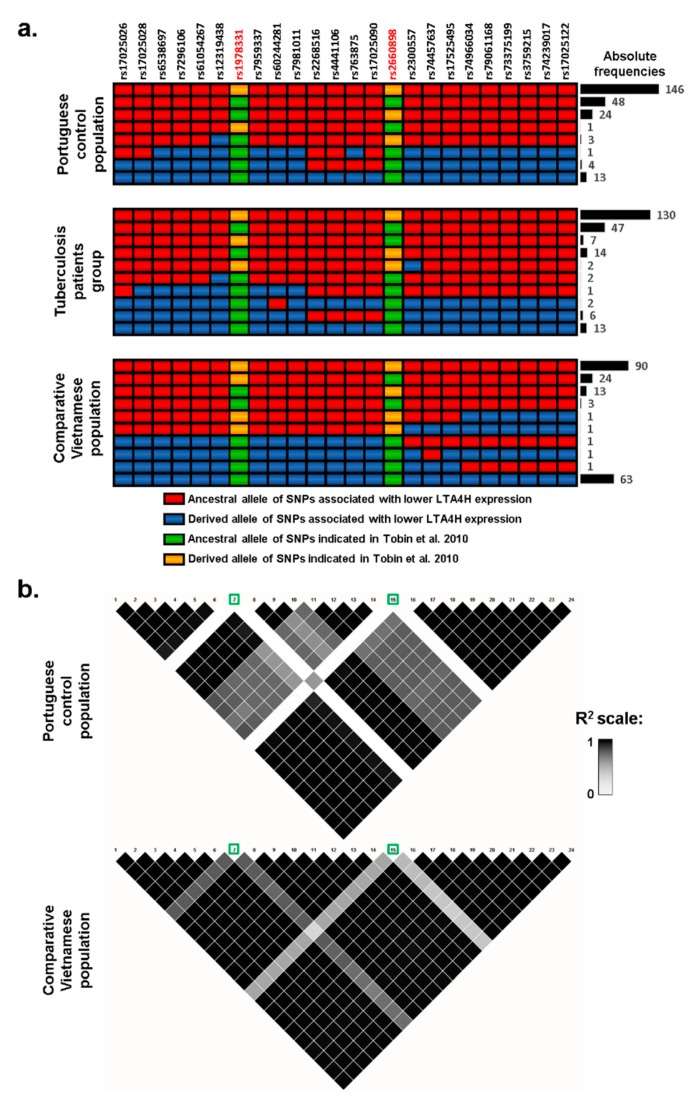
Haplotypic reconstruction and linkage map using 24 SNPs in the *lta4h* locus. We performed haplotypic reconstruction in TB patients, the control group, and a comparative Vietnamese group (**a**) and we performed linkage mapping using *r*^2^ in the Portuguese control group and the comparative Vietnamese group (**b**).

As lower and higher expression of LTA4H have previously been described as possible mechanisms for increased TB susceptibility [3], we explored possible associations with altered gene expression using a database of expression quantitative trait loci (eQTL), GTEx. Lower expression was associated with 22 SNPs (listed in Figure 2) which showed reductions between 36% and 50%, including the commonly typed SNP *rs17525495*, which previously showed an association with TB susceptibility in Vietnam [7] and other studies. An inspection of *rs17525495* and other eQTLs in our association data shows that, although frequencies of these SNPs are not significantly different between control and TB groups, they nevertheless have higher frequencies in patients (highlighted in Figure 1a,b), corresponding to an average ~30% frequency increase from 7.2% in controls to 9.3% in patients. For testing the distribution of haplotypes with these 22 eQTLs SNPs only in patients and controls, we again used a permutation test in PHASE. We obtained no significant difference between groups indicating that we could not reject the null hypothesis that control and patients belong to the same population set (*p* = 0.28).

We then compared haplotypes of patients and controls for the 22 eQTLs and *rs1978331*/*rs2660898* (previously associated with TB [3]). The 22 eQTLs are in strong linkage in the Portuguese population (Figure 2a), making it unclear which SNP or SNPs might be responsible for the lower expression levels we detected. At least one, *rs17025122*, proved to regulate LTA4H expression in vitro [12]. As noted above, these eQTLs had an average frequency of 7.2% in the control population and 9.3% in TB patients. If this ~30% increase represents a real positive association, we can estimate that we would need to sample over 2000 chromosomes from each group to obtain significance at a 5% level with a power of 80% (as applied in available online: https://clincalc.com), values which are unfortunately impossible for us to achieve for this cohort, particularly considering the limitations for sampling, which is restricted for the time period of the study by the stipulations of the ethical permission.

By contrast, these eQTLs have an average frequency of 35% in a Vietnamese population and a similar proportional increase in TB cases to that observed in our cohort (i.e., >30%) would be significant with only 200 chromosomes. Curiously, rs1978331/rs2660898 [3] are in linkage with these eQTLs in Vietnam (linked in 80.5% and 71%, respectively, of the estimated haplotypes), while this association is low in Portugal (20% and 28% of estimated haplotypes) (Figure 2a). This trend is clear also in the linkage maps, where *rs1978331*/*rs2660898* have *r*^2^ values of 0.6–0.8 in relation to the 22 eQTLs in the Vietnamese group, while the same *r*^2^ value is close to zero in the Portuguese setting (Figure 2b). Therefore, *rs1978331*/*rs2660898*, considering the possible association of the eQTLs with TB susceptibility, could provide false-positive associations, due to linkage to eQTLs in Vietnam but not in Europe, possibly explaining the different results in the different contexts [3,4,5,6]. Nevertheless, whether this is the case remains unclear, as the derived allele in one of the SNPs, *rs2660898*, shows substantially higher frequency in TB patients in Portugal (34% against 28% in the control population, showing the lowest *p*-value in the association analysis with covariates, Figure 1b), while the derived allele in *rs1978331* is actually more frequent in the control population (Figure 1).

## 4. Discussion

We have here performed the first genomic-scale analysis of the *lta4h* locus in groups of both TB patients and healthy controls. We compared 113 SNPs between the groups, but detected no significant difference, either considering individual SNPs (with and without adjustment for covariates), specific haplotypes or overall haplotype composition. The *rs17525495* SNP has previously been associated with both TB and bacterial meningitis susceptibility [7,13], and has been suggested as a candidate for extensive genotyping as a TB response predictor [14].

We detected that this SNP is in linkage with 21 other SNPs, all of them associated with decreased LTA4H expression in eQTL databases, in European, Asian and African [9,10] populations. In this study, derived alleles of *rs17525495* and the other eQTLs were more frequent in TB patients than controls, although that difference was not significant in the association studies we performed. The same situation has been seen in Mozambique for *rs17525495* [6], where there is again a nearly 30% increase of frequency of the derived allele between controls (11%) and patients (14%), without achieving significance in the analysis conducted. The complexity of the immune response might never lead to dramatic results for a single allele, and it may be that small differences in polymorphism frequencies can only be detected with very large cohorts [4].

In Asian cohorts, the situation is different, with *rs17525495* having shown a consistent association with TB and meningitis [7,13,15]. A possible explanation is that the derived allele frequencies of these eQTLs differ between Asian populations (~35%) and Africans and Europeans (only ~7–11%) [9,10], most probably the result of founder effects in the colonization of the different regions of the globe, as the most common haplotypes (containing the 22 detected eQTL SNPs and rs1978331/rs2660898) in each population are present both within and outside Africa. A negative effect on TB susceptibility caused by lower expression, due to the presence of these eQTLs, may, therefore, have much more impact in Asian populations (where *rs1978331*/*rs2660898* [3] are also linked to the same genetic background). *lta4h* genotyping might be important to establish the value of differential drug treatment [16] but one needs to demonstrate both the causality of the typed SNP or SNPs whose association can shift in different contexts and, importantly, their relevance in the population under study. Certainly, this does not mean that some SNPs with low frequency of the allele of interest in a given population, likely associated with TB susceptibility, are irrelevant in that population. Nevertheless, this scenario does imply a greater uncertainty regarding their importance in a public health context, given the associated costs of typing and their low frequency.

This study highlights the importance of a genomic approach, which allows us to take advantage of a full battery of statistical analyses and to thereby elucidate previously indiscernible patterns. This study paves the way for future work regarding the role of the LTA4H enzyme in susceptibility to TB, either focusing on genomic data or a larger set of relevant SNPs typed for the same populations/individuals, encompassing individuals from different origins. Such work would allow meta-analyses of variants and linkage patterns in different contexts and enable us to build robust evidence for the relevance and functional implication of a given variant.

## Figures and Tables

**Table 1:** Epidemiological and clinical characteristics of the tuberculosis (TB) and control groups.

	Control Population	Tuberculosis Patients	*p*-Value (*t*-Statistic Unless Stated)
n	120	112	
Age average	55.36	44.66	*p* < 0.0001 ^a^
Female/male ratio	60.83/39.17	34.82/65.18	*p* < 0.0001
**Co-morbidities:**			
Hypertension	25.00	7.14	*p* = 0.0002
Asthma	1.67	0.89	*p* = 0.6000
Bronchitis	1.67	1.79	*p* = 0.9442
COPD	1.67	3.57	*p* = 0.3634
Heart condition	5.83	3.57	*p* = 0.4192
Hepatitis	0.83	6.25	*p* = 0.0240
Silicosis	0	0.89	*p* = 0.3014
**Overall**	31.67	20.54	*p* = 0.0548
**Addictions:**			
Smoker/ex-smoker	35.83	58.93	*p* = 0.0565
Alcohol	0	8.04	*p* = 0.1906
Drugs	0.83	7.14	*p* = 0.2823
**Overall**	35.83	59.82	*p* = 0.0472

^a^ Obtained through a linear regression between phenotype and age.

## Supplementary Materials

Supplementary materials can be found at https://www.mdpi.com/2076-2607/7/12/650/s1, Table S1: Association analysis results of the 113 SNPs analysed in this study with and without covariates.
