# Supplementary material for: Association of Leukotriene A4 Hydrolase with Tuberculosis Susceptibility Using Genomic Data in Portugal
Source: Microorganisms. 2019 Dec 4;7(12):650. doi: 10.3390/microorganisms7120650 (PMC6956305; doi:10.3390/microorganisms7120650)
Supplement: Supplementary file 1 [file microorganisms-07-00650-s001.pdf]

Table S1. Association analysis results of the 113 SNPs analysed in this study with and without covariates.

SNP	Position in chromosome 12 (GRCh37)	Type of variation	Frequency in tuberculosis patients	Frequency in control population	*p-value* (no covariates)	*p-value* (with covariates)
rs117360077	96395377	intron variant	0.036	0.025	0.500	0.311
rs79858158	96396258	intron variant	0.152	0.154	0.943	0.506
rs17025026	96396410	intron variant	0.094	0.071	0.353	0.562
rs117664873	96396589	intron variant	0.058	0.054	0.856	0.854
rs17677343	96397081	intron variant	0.210	0.210	0.995	0.753
rs17025028	96398171	intron variant	0.098	0.071	0.271	0.407
rs2540500	96398828	intron variant	0.198	0.198	0.985	0.653
rs2660879	96399529	intron variant	0.058	0.058	0.989	0.736
rs2540499	96399869	intron variant	0.210	0.217	0.857	1.013
rs5020450	96399986	intron variant	0.152	0.146	0.857	0.411
rs17025033	96400460	intron variant	0.054	0.038	0.405	0.399
rs2540498	96400569	intron variant	0.214	0.229	0.700	0.934
rs2300559	96400832	intron variant	0.054	0.038	0.405	0.476
rs2660880	96401252	intron variant	0.058	0.063	0.840	0.622
rs2110762	96402534	intron variant	0.371	0.371	0.995	0.785
rs2072510	96403199	intron variant	0.384	0.379	0.916	0.703
rs116924183	96403353	intron variant	0.018	0.021	0.816	0.950
rs6538697	96403610	intron variant	0.098	0.075	0.343	0.502
rs7296106	96403894	intron variant	0.098	0.075	0.343	0.502
rs2540497	96404275	intron variant	0.210	0.217	0.857	0.830
rs2660900	96405077	intron variant	0.210	0.217	0.857	0.830
rs61054267	96405906	intron variant	0.098	0.075	0.343	0.502
rs2540496	96406503	intron variant	0.308	0.304	0.928	0.854
rs2540495	96406723	intron variant	0.313	0.300	0.770	0.774
rs12319438	96407086	intron variant	0.109	0.088	0.424	0.820
rs11108375	96408781	Intron/ splice region variant	0.054	0.038	0.405	0.399
rs2072512	96408976	intron variant	0.387	0.379	0.856	0.767

rs1978331	96409201	intron variant	0.378	0.388	0.840	0.919
rs12049939	96411397	intron variant	0.049	0.033	0.392	0.363
rs7959337	96411828	intron variant	0.098	0.075	0.343	0.502
rs2540494	96412338	intron variant	0.321	0.317	0.912	0.609
rs57218504	96413571	intron variant	0.032	0.029	0.895	0.639
rs57872417	96413683	intron variant	0.080	0.071	0.698	0.970
rs60244281	96413768	intron variant	0.089	0.075	0.545	0.661
rs75889755	96413977	intron variant	0.036	0.029	0.690	0.479
rs1990611	96413984	intron variant	0.371	0.361	0.838	0.637
rs7981011	96414337	intron variant	0.098	0.075	0.343	0.502
rs17677715	96414451	intron variant	0.156	0.154	0.951	0.424
rs2540493	96415842	intron variant	0.210	0.217	0.857	0.891
rs11108379	96415888	intron variant	0.049	0.038	0.538	0.560
rs2660838	96416383	intron variant	0.210	0.217	0.857	0.837
rs17677763	96416708	intron variant	0.156	0.154	0.951	0.424
rs2268516	96417199	intron variant	0.068	0.054	0.516	0.550
rs61937883	96419295	intron variant	0.104	0.135	0.308	0.648
rs4441106	96419455	intron variant	0.067	0.050	0.419	0.383
rs763875	96419941	intron variant	0.067	0.054	0.533	0.595
rs61937884	96420022	intron variant	0.018	0.042	0.134	0.346
rs763876	96420034	intron variant	0.040	0.033	0.695	0.541
rs763874	96420213	intron variant	0.228	0.246	0.664	0.915
rs17025090	96420953	intron variant	0.067	0.054	0.533	0.560
rs2660892	96420978	intron variant	0.145	0.188	0.222	0.532
rs2660893	96421921	intron variant	0.041	0.054	0.506	0.458
rs2660894	96422073	intron variant	0.041	0.054	0.492	0.484
rs2247570	96422377	intron variant	0.202	0.235	0.396	0.764
rs61937885	96423421	intron variant	0.152	0.154	0.943	0.508
rs2660895	96423447	intron variant	0.299	0.290	0.829	0.639
rs2660896	96423782	intron variant	0.393	0.378	0.746	0.598
rs2247330	96424122	intron variant	0.304	0.294	0.824	0.624
rs2247323	96424256	intron variant	0.313	0.294	0.667	0.542
rs2247313	96424447	intron variant	0.313	0.288	0.557	0.434

rs2247309	96424542	intron variant	0.308	0.292	0.701	0.540
rs2247304	96424665	intron variant	0.210	0.213	0.944	0.761
rs2660897	96425432	intron variant	0.384	0.378	0.898	0.712
rs11108381	96425491	intron variant	0.054	0.038	0.405	0.399
rs7956370	96425703	intron variant	0.161	0.150	0.750	0.328
rs2660898	96425997	intron variant	0.339	0.279	0.162	0.164
rs2540490	96426272	intron variant	0.321	0.286	0.404	0.394
rs2540489	96426502	intron variant	0.317	0.282	0.405	0.431
rs11108382	96426715	intron variant	0.076	0.038	0.072	0.171
rs2540488	96426745	intron variant	0.219	0.202	0.653	0.631
ss463060079	96427011	intron variant	0.022	0.025	0.850	0.535
rs2300557	96427293	intron variant	0.103	0.076	0.277	0.425
rs2246990	96427302	intron variant	0.313	0.282	0.466	0.520
rs145519475	96427468	intron variant	0.076	0.038	0.076	0.176
rs2246973	96427875	intron variant	0.335	0.282	0.215	0.245
rs2246972	96427889	intron variant	0.330	0.282	0.255	0.261
rs74457637	96428778	intron variant	0.094	0.075	0.437	0.678
rs17525495	96429377	5′ untranslated region variant	0.094	0.075	0.437	0.678
rs2540487	96429783	upstream gene variant	0.250	0.250	1.000	0.640
rs11108383	96429883	upstream gene variant	0.049	0.038	0.538	0.429
rs2660899	96430420	upstream gene variant	0.183	0.183	0.993	0.650
rs10777767	96430726	upstream gene variant	0.464	0.496	0.497	0.790
rs74966034	96430863	upstream gene variant	0.094	0.075	0.437	0.678
rs79061168	96430891	upstream gene variant	0.094	0.075	0.437	0.678
rs2540486	96431007	upstream gene variant	0.308	0.292	0.701	0.709
rs11108385	96431079	upstream gene variant	0.063	0.054	0.702	0.581
rs73375199	96431320	upstream gene variant	0.098	0.075	0.343	0.502
rs3759215	96431636	upstream gene variant	0.094	0.075	0.437	0.678
rs74239017	96432173	upstream gene variant	0.094	0.075	0.437	0.678
rs10777768	96432307	upstream gene variant	0.411	0.420	0.837	0.980
rs2660840	96432507	upstream gene variant	0.295	0.296	0.978	1.015
rs7966262	96433079	upstream gene variant	0.161	0.146	0.656	0.604

rs74389644	96433340	upstream gene variant	0.063	0.042	0.301	0.416
rs2540485	96433375	upstream gene variant	0.058	0.067	0.701	0.455
rs17025122	96433422	upstream gene variant	0.094	0.075	0.437	0.678
rs2540484	96433564	upstream gene variant	0.147	0.179	0.354	0.502
rs2540483	96434128	upstream gene variant	0.246	0.223	0.562	0.749
rs7971150	96434746	upstream gene variant	0.402	0.433	0.491	0.523
rs17025123	96434828	upstream gene variant	0.049	0.038	0.538	0.429
rs2540482	96434880	upstream gene variant	0.237	0.238	0.982	0.786
rs2540481	96434967	upstream gene variant	0.147	0.183	0.297	0.440
rs2540480	96435052	upstream gene variant	0.232	0.238	0.892	0.741
rs2540479	96435239	upstream gene variant	0.147	0.183	0.297	0.440
rs2887104	96435443	upstream gene variant	0.397	0.433	0.432	0.472
rs11108386	96435502	upstream gene variant	0.304	0.336	0.454	0.542
rs11830502	96435544	upstream gene variant	0.049	0.038	0.538	0.429
rs2367870	96435559	upstream gene variant	0.411	0.437	0.568	0.587
rs2367871	96435666	upstream gene variant	0.414	0.462	0.296	0.354
rs2540478	96435744	upstream gene variant	0.241	0.244	0.950	0.683
.	96435754	upstream gene variant	0.083	0.071	0.655	0.812
rs10860000	96435755	upstream gene variant	0.036	0.034	0.887	0.685
rs61937900	96435808	upstream gene variant	0.018	0.029	0.424	0.203
rs7314867	96436653	upstream gene variant	0.433	0.442	0.851	0.924
